# Liposomal encapsulation enhances and prolongs the anti-inflammatory effects of water-soluble dexamethasone phosphate in experimental adjuvant arthritis

**DOI:** 10.1186/ar3089

**Published:** 2010-07-19

**Authors:** Rebecca Anderson, Angels Franch, Margarida Castell, Francisco J Perez-Cano, Rolf Bräuer, Dirk Pohlers, Mieczyslaw Gajda, Alexandros P Siskos, Theodora Katsila, Constantin Tamvakopoulos, Una Rauchhaus, Steffen Panzner, Raimund W Kinne

**Affiliations:** 1 Experimental Rheumatology Unit, Department of Orthopedics, University Hospital Jena, Klosterlausnitzer Str. 81, 07607 Eisenberg, Germany; 2 Department of Physiology, Faculty of Pharmacy, University of Barcelona, Av. Joan XXIII s/n, 08028 Barcelona, Spain; 3 Institute of Pathology, University Hospital Jena, Ziegelmühlenweg, 07743 Jena, Germany; 4 Division of Pharmacology-Pharmacotechnology, Biomedical Research Foundation, Academy of Athens, Soranou Efesiou 4 street, 11527 Athens, Greece; 5 novosom AG, Weinbergweg 22, 06120 Halle/Saale, Germany

## Abstract

**Introduction:**

The objective of this study was to evaluate the efficacy of intravenous (i.v.) injection of liposomally encapsulated dexamethasone phosphate (DxM-P) in comparison to free DxM-P in rats with established adjuvant arthritis (AA). This study focused on polyethylene glycol (PEG)-free liposomes, to minimize known allergic reactions caused by neutral PEG-modified (PEG-ylated) liposomes.

**Methods:**

Efficacy was assessed clinically and histologically using standard scores. Non-specific and specific immune parameters were monitored. Activation of peritoneal macrophages was analyzed *via *cytokine profiling. Pharmacokinetics/biodistribution of DxM in plasma, synovial membrane, spleen and liver were assessed *via *mass spectrometry.

**Results:**

Liposomal DxM-P (3 × 1 mg/kg body weight; administered intravenously (i.v.) on Days 14, 15 and 16 of AA) suppressed established AA, including histological signs, erythrocyte sedimentation rate, white blood cell count, circulating anti-mycobacterial IgG, and production of interleukin-1beta (IL-1β) and IL-6 by peritoneal macrophages. The suppression was strong and long-lasting. The clinical effects of liposomal DxM-P were dose-dependent for dosages between 0.01 and 1.0 mg/kg. Single administration of 1 mg/kg liposomal DxM-P and 3 × 1 mg/kg of free DxM-P showed comparable effects consisting of a partial and transient suppression. Moreover, the effects of medium-dose liposomal DxM-P (3 × 0.1 mg/kg) were equal (in the short term) or superior (in the long term) to those of high-dose free DxM-P (3 × 1 mg/kg), suggesting a potential dose reduction by a factor between 3 and 10 by liposomal encapsulation. For at least 48 hours after the last injection, the liposomal drug achieved significantly higher levels in plasma, synovial membrane, spleen and liver than the free drug.

**Conclusions:**

This new PEG-free formulation of macrophage-targeting liposomal DxM-P considerably reduces the dose and/or frequency required to treat AA, with a potential to enhance or prolong therapeutic efficacy and limit side-effects also in the therapy of rheumatoid arthritis. Depot and/or recirculation effects in plasma, inflamed joint, liver, and spleen may contribute to this superiority of liposomally encapsulated DxM-P.

## Introduction

Rheumatoid arthritis (RA) is a systemic disorder of unknown etiology characterized by chronic inflammation and symmetric, progressive destruction of arthritic joints. The abundance and activation of macrophages (Mφ) in the inflamed synovial membrane significantly correlates with the severity of RA [1,2]. In addition, activation of the monocytic lineage extends to systemic parts of the mononuclear phagocyte system [3-7]. Thus, selective counteraction of Mφ activation is a promising approach to diminish local and systemic inflammation or to prevent irreversible joint damage.

In addition to disease-modifying anti-rheumatic drugs (DMARDs) and to recently introduced biologicals (for example, antibodies against tumor necrosis factor- (TNF)-α or soluble TNF-α-receptors, [8-14]), anti-inflammatory glucocorticoids are still frequently employed to bridge the gap before the onset of action of DMARDs [15-17] and to improve the therapeutic control of RA. Due to their unequalled efficacy, bridging application and wide indication range (for example, renal failure, pregnancy), glucocorticoids remain valuable therapeutic tools. However, concerns about long-term side effects, such as Cushing syndrome or bone demineralization, strongly emphasize the need for safer treatment modalities. Specific targeting of glucocorticoids to phagocytic cells by liposomal encapsulation potentially increases drug efficacy and longevity while minimizing side-effects [18,19].

Previous studies have demonstrated good therapeutic efficacy of water-soluble prednisolone in neutral polyethylene glycol-modified (PEGylated) liposomes in animal models of arthritis and multiple sclerosis [20-23]. However, evidence has emerged that repeated injections can result in the generation of anti-PEG antibodies, emphasizing the need for a PEG-free liposomal formulation [24-27].

In this study, water-soluble dexamethasone phosphate (DxM-P) was encapsulated in a novel, non-PEGylated liposome formulation (Micromethason). All lipids in this formulation have market approval, thereby minimizing the risk of lipid-associated toxicity. The efficacy of liposomal DxM-P was evaluated in rat adjuvant arthritis (AA), a severe animal model characterized by histopathological similarities to RA, including both systemic and local features of inflammation [28]. The effects of treatment with free DxM-P or different doses of liposomally-encapsulated DxM-P were also evaluated, in order to assess the increase of therapeutic potency by encapsulation. In addition, a pharmacokinetics and biodistribution study of DxM was carried out following administration of liposomal DxM-P or free DxM-P.

## Materials and methods

### Micromethason preparation

Micromethason liposomes were prepared by Novosom AG (Halle, Germany) from 1,2-dipalmitoyl-sn-glycero-3-phosphocholine (DPPC), 1,2-dipalmitoyl-sn-glycero-3-(phosphor-rac-(1-glycerol))(sodium salt) (DPPG) and cholesterol (50:10:40 mol %; all lipids with market approval) using the lipid film extrusion method [29]. The lipid film was hydrated with DxM-P (25 mg/ml in phosphate-buffered saline (PBS), pH 7.5) and the resulting vesicles were extruded through 400 nm membranes. Non-encapsulated DxM-P was removed by gel filtration. Particle size (283 to 310 nm) and polydispersity (≤0.3) were determined by dynamic light scattering. The drug/lipid ratio was 40 μg/μmol (determined as in [30]) and the concentration was adjusted to 500 μg DxM-P/ml.

The liposomes were selected for tolerability *in vivo *and their ability to target cells of the macrophage-phagocyte system while avoiding unwanted sites such as the liver parenchyme. The latter was achieved by using rather large particles, which cannot penetrate across the 150 nm fenestrations of liver endothelium [31]. The cellular targeting to cells of the macrophage-phagocyte system relates to the negative surface charge of the liposomes [32].

### Adjuvant arthritis

#### Animals

Female Lewis rats (seven to eight weeks of age) were obtained from Harlan (Barcelona, Spain) and housed three to four per cage under standard conditions, with food and water *ad libitum *and a 12-hour-light/12-hour-dark cycle. The animals were allowed two weeks to adjust to the housing conditions prior to the initiation of studies. The experiments were performed in accordance with the Institutional Guidelines for the Care and Use of Laboratory Animals (Ethics Committee for Animal Experimentation, Universities of Barcelona and Jena).

#### Experimental design

**Figure 1:**
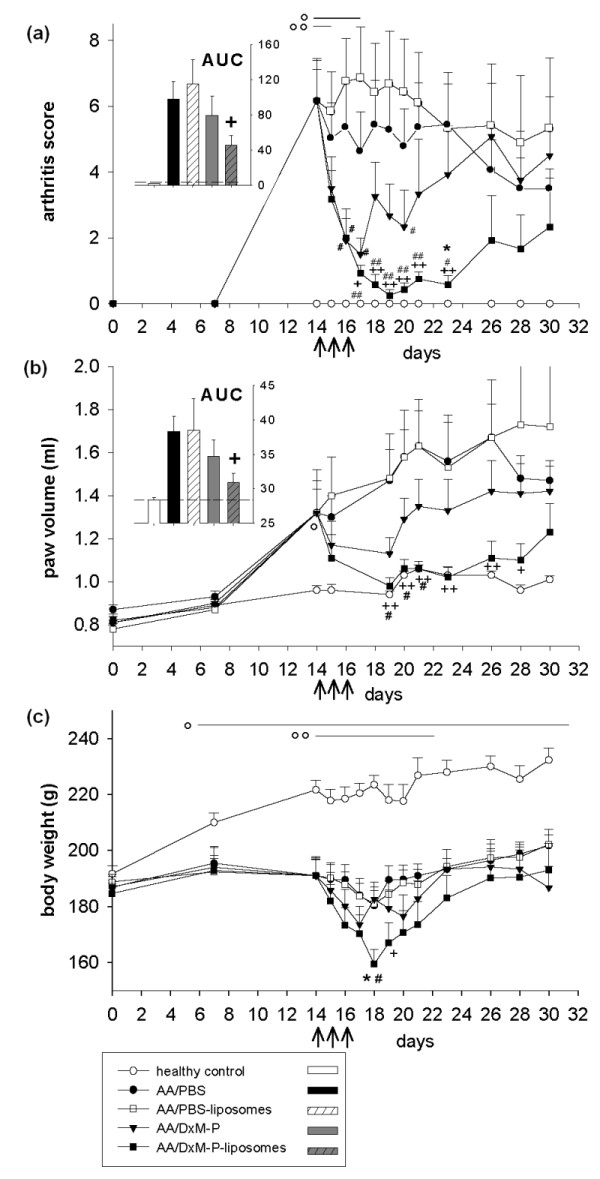
**Clinical effects of free or liposomal DxM-P in adjuvant arthritis (Experiment 1)**. Effect of treatment with PBS-liposomes, free DxM-P (3 × 1 mg/kg; Days 14, 15 and 16), or liposomal DxM-P (3 × 1 mg/kg; Days 14, 15 and 16) on arthritis score **(a)**, paw volume **(b)**, and body weight **(c)**; all arthritic groups were normalized to the same mean value for each parameter on Day 14 (*n *= 6 each). The inserts show the area under the curve (AUC) from Day 0 to Day 30 for the arthritis score (a) and the paw volume (b); dashed line: healthy control levels. For all treatment groups: + *P *≤ 0.05, ++ *P *≤ 0.01 *vs*. AA/PBS; # *P *≤ 0.05, ## *P *≤ 0.01 *vs*. AA/PBS-liposomes; * *P *≤ 0.05 *vs*. AA/DxM-P; For AA/liposomal DxM-P: o *P *≤ 0.05, oo *P *≤ 0.01 *vs*. healthy controls; all Mann Whitney U-test; ↑ I. v. administration of PBS, PBS-liposomes, free DxM-P, or liposomal DxM-P.

**Figure 2:**
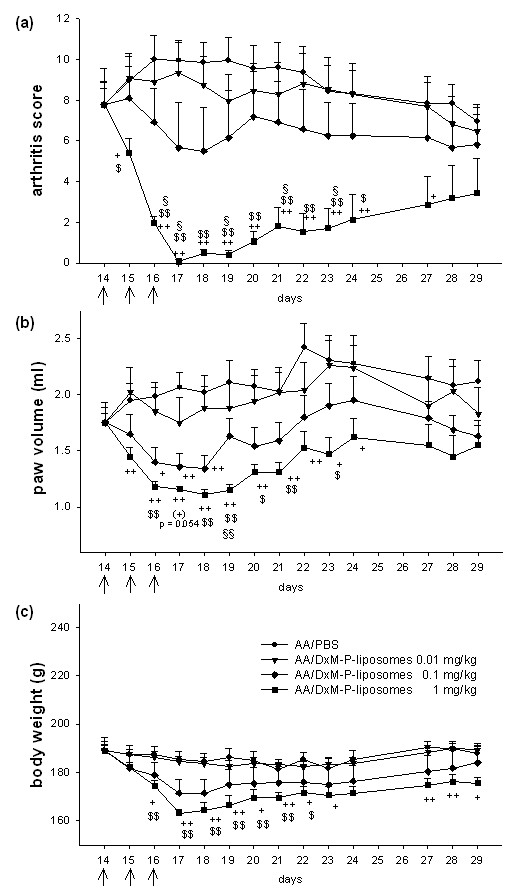
**Dose-dependence of liposomal DxM-P in adjuvant arthritis (Experiment 4)**. **(a-c)**: Dose-response of treatment with liposomal DxM-P (3 × 1 mg/kg, 3 × 0.1 mg/kg, or 3 × 0.01 mg/kg); all groups were normalized to the same mean value for each parameter on Day 14 (*n *= 6 each). + *P *≤ 0.05, ++ *P *≤ 0.01 *vs*. AA/PBS; $ *P *≤ 0.05, $$ *P *≤ 0.01 *vs*. AA/DxM-P-liposomes 0.01 mg/kg; § *P *≤ 0.05, §§ *P *≤ 0.01 *vs*. AA/DxM-P-liposomes 0.1 mg/kg, * *P *≤ 0.05 *vs*. AA/DxM-P 1 mg/kg; all Mann Whitney U-test. ↑ administration of PBS or liposomal DxM-P.

On Day 0, rats were injected intradermally (i.d.) into the tail base with 0.5 mg of heat-killed *Mycobacterium butyricum *(Mb) (Difco, Detroit, MI, USA) in 0.1 ml of liquid vaseline as previously published [33]. Only animals developing clear signs of arthritis on Day 14 (arthritis score ≥2) were used for further studies (*n *= 6 for all groups). On Days 14, 15 and 16 of AA, anesthetized rats were treated intravenously (i.v.; tail vein) with 1 mg/kg body weight liposomal DxM-P (AA/DxM-P-liposomes; usually 250 μg/250 g body weight). Arthritic controls were injected with either 3 × 1 mg/kg body weight of free DxM-P (AA/DxM-P), an equal volume of PBS (AA/PBS), or PBS-containing liposomes (AA/PBS-liposomes). The healthy controls were not immunized and did not receive any treatment. For dose-response studies, animals were treated with 3 × 0.01 mg/kg, 3 × 0.1 mg/kg, or 3 × 1 mg/kg of liposomal or free DxM-P. In addition, one group of rats received a single dose of 1 mg/kg of liposomal DxM-P on Day 14 of AA. In total, three different arthritis studies were performed, that is, Experiment 1 (treatment study; see Table 1 and Figure 1), Experiment 2/3 (two different animal subgroups of the same treatment study containing both groups with triple and single treatment with liposomal DxM; Table 1), and Experiment 4 (dose response; see Table 2 and Figure 2).

**Table 1:** Reduction of clinical parameters by liposomal dexamethasone phosphate on Days 19 and 28 of adjuvant arthritis

Experiment 1	Arthritis score		Paw volume	
		
*Treatment*	Day 19	Day 28	Day 19	Day 28
*AA/PBS*	--	--	--	--
*AA/PBS-liposomes*	+23% ± 29.4%	+36% ± 56.5%	+ 2% ± 38.8%	+47% ± 62.3%
*AA/DxM-P*	-51% ± 17.8%	+ 4% ± 41.7%	-65% ± 14.0%	-13% ± 26.4%
*AA/liposomal DxM-P*	-95% ± 3.2%^++ ##^	-54% ± 28.7%	-92% ± 7.4%^++ #^	-73% ± 17.3% ^+^

**Experiment 2/3**	**Arthritis score**		**Paw volume**	
		
***Treatment***	**Day 19**	Day 29*	**Day 19**	Day 29*

*AA/PBS*	--	--	--	--
*AA/PBS-liposomes*	+15% ± 19.8%		+23% ± 20.3%	
*AA/DxM-P*	-67% ± 16.4% ^+ #^		-52% ± 21.8% ^+ #^	
*AA/liposomal DxM-P*	-90% ± 5.0%^++ ##^		-87% ± 6.6.%^++ ##^	

AA/liposomal DxM-P 1× *	-48% ± 23.9%	-15% ± 29.6%	-63% ± 24.6%	-35% ± 22.6%

Reduction of arthritis score and paw volume on Day 19 (maximal efficacy of therapy) and Day 28 of AA after treatment with PBS-liposomes, free DxM-P (3 × 1 mg/kg; Days 14, 15 and 16), or liposomal DxM-P (3 × 1 mg/kg; Days 14, 15 and 16) compared to AA/PBS (Experiment 1 corresponds to the data in Figure 1). For comparison, the effects of treatment with single-dose liposomal DxM-P (1 × 1 mg/kg; Day 14; Experiment 3) are shown. * results from a separate subgroup of animals (named Experiment 3 for clarity) from the same arthritis study with similar disease severity on Days 19 and 20 as the other subgroup of animals (named Experiment 2).

Data are expressed as means ± standard error of the mean.

+ *P *≤ 0.05, ++ *P *≤ 0.01 *vs*. AA/PBS; # *P *≤ 0.05, ## *P *≤ 0.01 *vs*. PBS-liposomes.

**Table 2:** Dose-dependent reduction of clinical parameters by liposomal dexamethasone phosphate on Days 19 and 28 in adjuvant arthritis

Experiment 4		Arthritis score		Paw volume	
			
*Treatment*		Day 19	Day 28	Day 19	Day 28
*AA/PBS*		--	--	--	--
*AA/DxM-P*	*0.01 mg/kg*	-10% ± 9.3%	+ 6% ± 11.9%	-18% ± 12.9%	-12% ± 8.4%
*AA/DxM-P*	*0.1 mg/kg*	-25% ± 6.9%	-4% ± 10.3%	-34% ± 10.1%	+ 9% ± 27.0%
*AA/DxM-P*	*1.0 mg/kg*	-69% ± 12.5% ^+^	-27% ± 16.4%	-71% ± 10.4% ^+^	-23% ± 16.0%
*AA/liposomal DxM-P*	*0.01 mg/kg*	-20% ± 14.0%	-13% ± 18.5%	-21% ± 21.1%	-5% ± 26.7%
*AA/liposomal DxM-P*	*0.1 mg/kg*	-38% ± 23.7%	-28% ± 16.9%	-44% ± 15.4%	-36% ± 12.2%
*AA/liposomal DxM-P*	*1.0 mg/kg*	-96% ± 2.0% ^++ $$ § ^*	-59% ± 20.0%	-86% ± 5.1% ^++ $$ §§^	-58% ± 18.0%

Reduction of arthritis score and paw volume on Day 19 (maximal efficacy of therapy) and Day 28 of AA after treatment with free DxM-P (3 × 1 mg/kg, 3 × 0.1 mg/kg, or 3 × 0.01 mg/kg) or liposomal DxM-P (3 × 1 mg/kg, 3 × 0.1 mg/kg, or 3 × 0.01 mg/kg) compared to AA/PBS (Experiment 4 corresponds to the data in Figure 2).

Data are expressed as means ± standard error of the mean.

+ *P *≤ 0.05, ++ *P *≤ 0.01 *vs*. AA/PBS; $$ *P *≤ 0.01 *vs*. AA/liposomal DxM-P 0.01 mg/kg; § *P *≤ 0.05, §§ *P *≤ 0.01 *vs*. AA/liposomal DxM-P 0.1 mg/kg; * *P *≤ 0.05 *vs*. AA/DxM-P 1 mg/kg.

#### Clinical parameters

Clinical signs of AA were assessed by measuring body weight (precision scale), arthritis score (blinded grading of each paw from 0 to 4 according to the extent of edema and erythema of the periarticular tissue, as well as the deformation of the joint; maximal score per animal: 16), and hind paw volume (water plethysmometer; LI 7500 Letica, Spain). Except for the healthy controls, all groups were normalized to the same mean value for each parameter on Day 14; the volume of both hind paws was averaged.

#### Histology

Animals were sacrificed on Day 21 of AA (that is, shortly after the maximal efficacy of therapy) and the hind paws were removed, skinned, fixed in 3% paraformaldehyde, decalcified, and embedded in paraffin. Microtome sections (3 to 5 μm) of decalcified joints were deparaffinized and stained with hematoxylin and eosin (HE). Severity of arthritis was examined by blinded grading of acute inflammation (graded from 0 (absent) to 9 (very severe) according to the magnitude of granulocyte infiltration and exudates) and chronic inflammation (graded from 0 to 9 according to the magnitude of synovial hyperplasia, mononuclear infiltration, and affection of periarticular structures), as well as joint and bone destruction (each graded from 0 to 3). Parameters were semi-quantitatively judged by an experienced pathologist (MG).

#### Erythrocyte sedimentation rate (ESR), white blood cell count (WBC), and differential leukocyte count

Blood samples were obtained by cardiac puncture of anesthetized rats on Day 21 of AA. To determine the ESR, 1 ml of tri-sodium citrate-treated blood was left to sediment for one hour in capillaries (Tapval™ tubes, Aquisel, Barcelona, Spain). The WBC was determined automatically using a Coulter Counter JT hemocytometer (Hialeah, FL, USA) calibrated for rat blood, and the differential white blood count was obtained by manual enumeration of May-Grünwald-Giemsa-stained blood cell smears.

#### Determination of serum anti-Mb antibodies

Anti-Mb immunoglobulin G (IgG) in rat sera (diluted 1:1.000 and 1:10.000 in BSA/PBS/0.05% Tween) was measured by ELISA using Mb-coated microplates (3 μg/ml of mycobacterial protein) as previously published [33,34].

#### Delayed-type hypersensitivity

To assess the delayed-type hypersensitivity (DTH), 50 μl Mb (1.5 mg/ml of PBS) and 50 μl PBS, respectively, were injected intradermally into the left and right ear on Day 19 (that is, 48 hours prior to sacrifice). The ears of anesthetized rats were cut, weighed, and the DTH was expressed as the x-fold enlargement in comparison to the contra-lateral control ear.

#### Isolation of spleen and lymph node lymphocytes

Spleen and popliteal lymph nodes (the latter as draining node of the arthritic hind paw; pop LN) were obtained on Day 21. Spleen cell suspensions were prepared by passing the samples through stainless steel sieves and eliminating the erythrocytes by hypotonic lysis (consecutive resuspension in 1 ml sterile PBS, 8 ml H_2_O, and 1 ml 10 × PBS). Pop LN were passed through stainless steel sieves and sterile gauze to eliminate connective tissue. After centrifugation (10 minutes; 600 × *g*), cell suspensions were resuspended in RPMI 1640 plus 10% FBS, and the lymphocytes counted using Fast Read^® ^chamber slides under fluorescence light after vital staining with acridine orange and ethidium bromide.

#### Isolation and stimulation of peritoneal Mφ (PM)

PM were harvested on Day 21 by peritoneal lavage with 40 ml ice-cold PBS, centrifuged, washed, and resuspended in RPMI 1640 containing 10% FCS and 5% gentamycine. Adherent Mφ (1 × 10^6 ^cells per well of 12-well plates after adherence for two hours at 37°C) were stimulated for 24 hours with LPS (1 μg/ml; *E coli*; Serotype 0111:B4; Sigma-Aldrich, Munich, Germany). Cytokines were analyzed in cell-free supernatants by ELISA using rat TNF-α and rat IL-6 (BD Biosciences Pharmingen, San Diego, CA, USA) or rat IL-1βkits (R&D Systems, Minneapolis, MN, USA).

#### Pharmacokinetics/biodistribution

The analysis of DxM concentrations in different body compartments was performed as previously published with slight modifications [35,36].

#### Materials

HPLC-grade acetonitrile (AcCN) was purchased from Fisher Scientific (Loughborough, UK). HPLC-grade ammonium acetate (NH_4_Ac) and HPLC-grade formic acid (FA) were purchased from Sigma-Aldrich (Athens, Greece). Purified water was produced using a Barnstead NANOpure Diamond water system from Barnstead International (Dubuque, IA, USA). DxM and prednisolone were purchased from Riedel-de Haen (ChemiLab, Athens, Greece).

#### Preparation of standards and calibration curve for method validation

Prednisolone was used as an internal standard (IS) for the quantitative analysis of DxM. Stock solutions of both DxM (5 to 10,000 ng/ml) and prednisolone (1 μg/ml) were prepared in methanol. Calibration curves (2 to 4,000 ng/ml; limit of quantification 2 ng/ml) and quality control samples (800 ng/ml) in plasma were prepared daily by adding 20 μl of DxM and 20 μl of IS solutions into 50 μl of plasma. For tissue analysis, calibration curves were constructed daily in a similar manner by adding standards of DxM and IS into 100 μl of tissue homogenate (liver, spleen or synovial membranes) at concentrations of 2.5 to 2,000 ng/g (limit of quantification 2.5 ng/g). Calibration curves were constructed using a linear fit (1/x^2 ^weighing) in the concentration range of 2 to 4,000 ng/ml for plasma and 2.5 to 2,000 ng/g for the tissues. The accuracy in the concentration range was ±20% (that is, target concentrations were within 20% of nominal values). The recovery of DxM was evaluated in rat plasma (96% at a concentration of 200 ng/ml) and in rat liver (91% at a concentration of 1,000 ng/g).

#### Tissue/organ sampling

Heparinized plasma samples were obtained by retro-orbital puncture and subsequent centrifugation 30 minutes, 2 hours, 8 hours, and 24 hours after the first administration of free or liposomal DxM-P (1 mg/kg each; 1^st ^administration = time point 0 for pharmacokinetic results; Day 14 of AA), as well as 72 hours, 96 hours, and 9 days after the first dose (these animals received a total of three drug administrations). Synovial membrane, spleen, and liver where also obtained 72 hours, 96 hours, and 9 days after the first injection (total of three doses); all samples were immediately shock frozen in liquid nitrogen.

#### Sample preparation

##### Plasma

Prior to the extraction procedure, 20 μl of the IS solution was added to 50 μl of mouse plasma. Samples were prepared for LC/ESI-MS/MS analysis by protein precipitation by addition of 100 μl acetonitrile:water (50:50), followed by stepwise addition of (2 × 150 μl) of ice-cold acetonitrile and brief vortexing and sonication. The samples were subsequently centrifuged (12,000 × *g*) for 15 minutes, and the supernatant was transferred into glass tubes and evaporated in a SpeedVac SPD1010 (Thermo Fisher Scientific Inc., Waltham, MA, USA) for 70 minutes at 50°C. Until analyzed, samples were stored at -20°C. An aliquot of 200 μl of mobile phase of the initial chromatographic conditions A/B 95/5 was added to each sample, vortexed, and transferred onto a 96-well plate for analysis by LC/ESI-MS/MS.

##### Tissues/organs

Synovial membrane, spleen, or liver homogenates were prepared as follows: tissues were washed once with 5 ml of water. Water was decanted after washing and 4 ml of 0.1% formic acid in water per g of tissue were added to the respective tissues. The mixture was homogenized in an Ultra-Turrax T8 homogenizer (IKA^®^-Werke, Germany) and subsequent analysis was carried out immediately after homogenization. Prior to the extraction of DxM from tissue homogenates, 20 μl of the IS solution was added to 100 μl of tissue homogenate. Samples were prepared for LC/ESI-MS/MS analysis by protein precipitation *via *addition of 200 μl acetonitrile:water (50:50), followed by stepwise addition of (2 × 300 μl) of ice-cold acetonitrile and brief vortexing/sonication. The samples were subsequently centrifuged for 15 minutes, and the supernatant was transferred into glass tubes and evaporated in a SpeedVac SPD1010 for 70 minutes at 50°C. Samples were stored at -20°C. An aliquot of 100 μl of mobile phase of the initial chromatographic conditions A/B 95/5 was added to each sample, vortexed, and transferred onto a 96-well plate for analysis by LC/ESI-MS/MS.

#### LC-MS/MS analysis

HPLC was performed with an Agilent 1100 Series system (Agilent Technologies, Waldbrown, Germany) equipped with a binary pump, autosampler, vacuum degasser, and temperature-controlled column compartment. The mobile phase consisted of solvents A: 10% acetonitrile, 90% water, 2 mM ammonium acetate, 0.1% formic acid and B: 90% acetonitrile, 10% water, 2 mM ammonium acetate, 0.1% formic acid. A Waters Symmetry C8 3.5 μm, 2.1 × 50 mm column (Waters Corporation, Milford, MA, USA) was used at a flow rate of 0.3 ml/minute. The analytical gradient profile was as follows (minute/% of B): 0/5, 1/5, 6/50, 9/50, 11/5, 14/5, total run time 14 minutes. The typical injection volume was 10 μl. Mass spectrometry was performed on an API 4000 QTRAP™ LC-MS/MS system fitted with a TurboIonSpray source and a hybrid triple quadrupole/linear ion trap mass spectrometer (Applied Biosystems, Concord, ON, Canada). The instrument was operated in positive ion mode with an IonSpray voltage of 5500 V, a source temperature of 550°C, curtain gas (nitrogen) at 20, collision gas (nitrogen) at 5, ION source gas 1 (air) at 40 and Ion source gas 2 (air) at 45 (all arbitrary units). Quantification was performed in the MRM positive mode. For the detection of DxM (MW: 392.5), the transitions of *m/z *393.6 → 373.4, *m/z *393.6 → 355.5 and 393.6 → 337.4 were optimized by adjustments of collision energy, declustering potential and dwell time. IS (prednisolone MW: 360.4) was monitored using the MRM transition of *m/z *361.6 → 343.3. The lower limit of quantification was 2.0 ng/ml in plasma and 2.5 ng/g in tissue.

The analytical procedure focussed on rigorous and validated quantification of DxM as the pharmacologically relevant analyte. In selected spleen samples, DxM-P detection was also accomplished in the negative ionization mode following assessment of: i) selectivity of the analysis for DxM and DxM-P; and ii) autosampler stability.

### Statistics

Differences among groups were analyzed using the Mann Whitney U-Test (*P *≤ 0.05), correlations among variables using the Spearman rank correlation (both SPSS 13.0™; SPSS Inc.; Chicago, IL, USA).

## Results

### Clinical effects of Micromethason therapy in AA

Short term i.v. treatment of established AA with liposomal DxM-P on Days 14, 15 and 16 (3 × 1 mg/kg body weight) resulted in a strong, significant, and long-lasting suppression of arthritis score and paw volume to almost normal levels (Figure 1a, b; Table 1). On Day 28, that is, two weeks after the initiation of treatment, liposomal DxM-P still reduced the arthritis score by 54% and the paw volume by 73% (Table 1). Despite a significant, but transient drop of the body weight after therapy with liposomal DxM-P (Figure 1c), the treatment was well tolerated by the rats, which showed an active and mobile behavior from Day 15 onwards. In contrast, the same dosage of free DxM-P (3 × 1 mg/kg) only transiently reduced the arthritis score by 51% and the paw volume by 65%, with a strong rebound starting two days after the end of therapy (Figure 1a, b; Table 1). PBS-liposomes were ineffective or even induced a transient, numerical increase of the clinical signs (Figure 1a, b; Table 1).

Similar results were obtained in two independent repeats (Table 1; Experiments 2/3 and Table 2; Experiment 4), confirming the validity of the data.

Interestingly, reduction of the area under the curve (AUC from Day 0 to Day 30) by liposomal DxM-P reached almost 54% for the arthritis score (*P *≤ 0.05; 19% with free DxM-P) and 74% for the paw swelling (*P *≤ 0.05; 36% with free DxM-P; see inserts in Figure 1a, b).

### Dose-response experiments

The effects of liposomal DxM-P were dose-dependent (Figure 2a-c; Table 2), that is, inhibition of arthritis score and paw swelling was significantly stronger after administration of high-dose liposomal DxM-P than after low-dose and medium-dose liposomal DxM-P (Figure 2a, b; Table 2).

Efficacy differences between liposomal and free DxM-P were dose-dependently potentiated. Compared to free DxM-P, the reduction of the arthritis score on Day 19 was 10% higher with low-dose liposomal DxM-P, 13% higher with medium-dose DxM-P, and 27% higher with high-dose DxM-P (Table 2). The differences between high-dose liposomal DxM-P and high-dose free DxM-P were significant for the reduction of arthritis score (*P *≤ 0.05, Days 16 to 19), paw volume (*P *≤ 0.05, Day 18), but also the reduction of body weight (*P *≤ 0.05, Days 17 and 18; data not shown).

### Single-dose liposomal DxM-P

Single-dose therapy with liposomal DxM-P (1 mg/kg) on Day 14 caused a significant, but short-lasting reduction of arthritis score (*P *≤ 0.05 *vs*. AA/PBS on Day 15; *P *≤ 0.08 on Days 16 and 17) and paw volume (*P *≤ 0.05 *vs*. AA/PBS on Day 15; *P *≤ 0.06 on Days 16, 17 and 19; data not shown). The magnitude of the effects on Days 19 and 29 were similar or even superior to those after therapy with three × free DxM-P in the same experimental group of animals (1 mg/kg; Table 1; experimental subgroups named Experiment 2 and 3).

**Figure 3:**
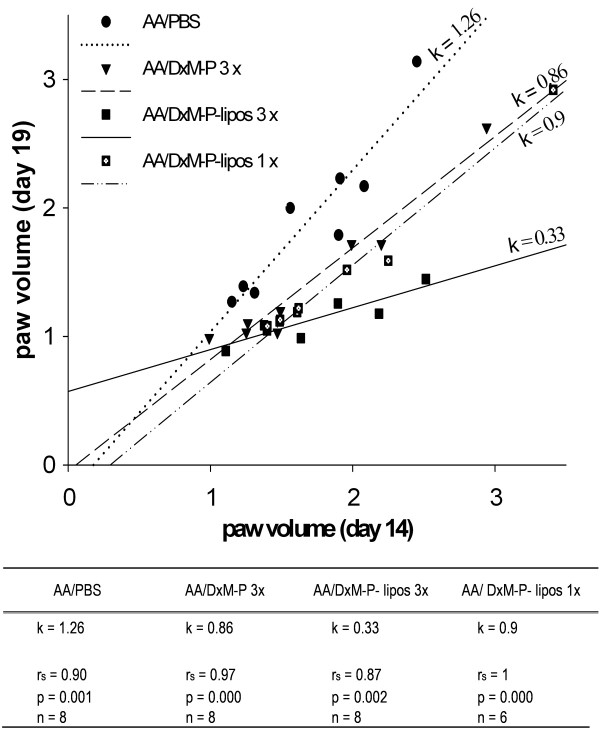
**Correlation between the paw volume on Days 14 and 19 during the time course of adjuvant arthritis**. Correlation (Spearman rank test, rs) between the paw volume on Day 19 (maximal efficacy of treatment) and Day 14 (prior to treatment) of individual rats treated with PBS (3 ×; Days 14, 15 and 16; *n *= 8), free DxM-P (3 × 1 mg/kg; Days 14, 15 and 16; *n *= 8), or liposomal DxM-P (3 × 1 mg/kg; Days 14, 15 and 16; *n *= 8; or 1 × 1 mg/kg; Day 14; *n *= 6). The solid, dashed, and dotted lines are the regression lines, k represents the regression gradient.

In all groups, the paw volume on Day 19 showed a significant, positive correlation with the volume before treatment (Day 14; *P *≤ 0.005, rs ≥ 0.87; *n *= 30; Figure 3). The regression line gradient for rats treated three × with high-dose liposomal DxM-P was k = 0.33, whereas that for PBS-treated rats was 1.26. These results show that three × administration of liposomal DxM-P strongly controlled the swelling even in the case of established, severe AA. The regression line gradient of three × treatment with liposomal DxM-P was smaller than that of one × treatment with liposomal DxM-P, indicating the dose-dependent effect on arthritis. Rats treated one × with liposomal DxM-P or three × with free DxM-P showed similar gradients (k = 0.9 and 0.86, respectively) and thus comparable therapeutic efficacy (Figure 3).

### Histological results

**Figure 4:**
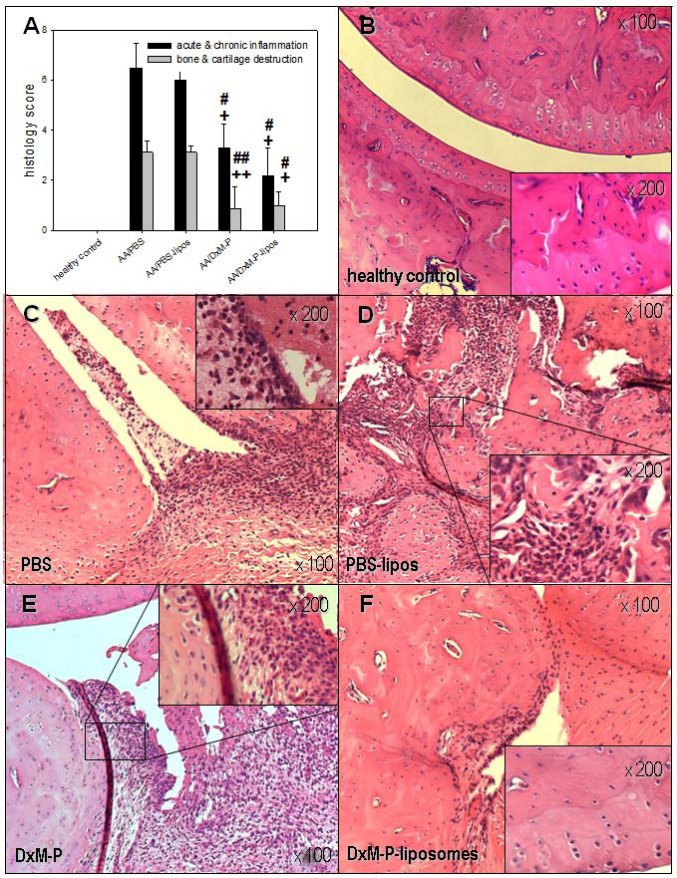
**Histological effects of liposomal DxM-P in adjuvant arthritis (Day 21; Experiment 2/3)**. Inflammation and destruction score **(a) **and images of HE-stained joints sections from healthy controls **(b) **or arthritic rats after treatment with PBS **(c)**, PBS-liposomes **(d)**, free DxM-P **(e)**, or liposomal DxM-P **(f) **(for details see Figure 1). + *P *≤ 0.05, ++ *P *≤ 0.01 *vs*. AA/PBS; # *P *≤ 0.05, ## *P *≤ 0.01 *vs*. AA/PBS-liposomes; all Mann Whitney U-test.

On Day 21, untreated arthritic animals showed massive infiltration with acute/chronic inflammatory cells and synovial hyperplasia, as well as marked loss of bone and cartilage (Figure 4c). Both free DxM-P and liposomal DxM-P significantly inhibited acute granulocytic inflammation and joint destruction, although some chronic infiltration of mononuclear cells, synovial hyperplasia, and deformation persisted (Figure 4a, e-f). Signs of chronic and especially acute inflammation were reduced to a somewhat greater extent by liposomal than by free DxM (data not shown).

### Hematological results

#### ESR

**Figure 5:**
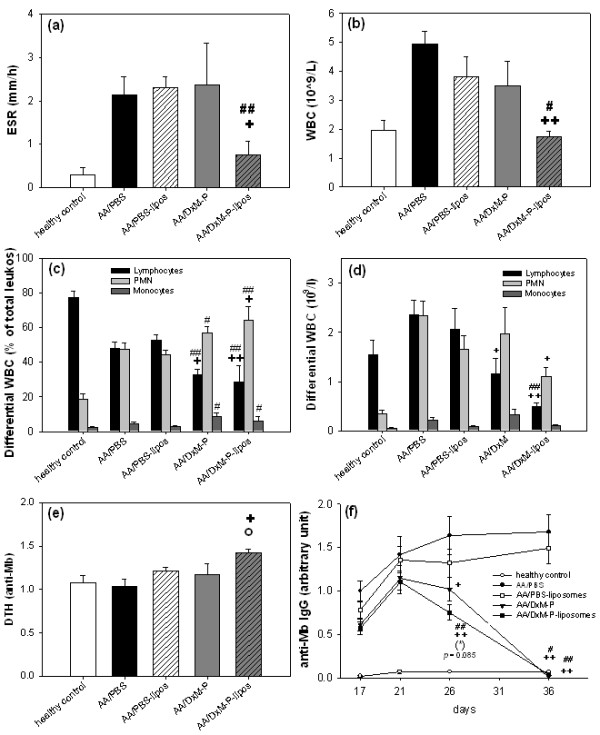
**Hematological parameters (Day 21) and anti-Mb IgG response (Days 17 to 36) in adjuvant arthritis (Experiment 2/3)**. Erythrocyte sedimentation rate (ESR) **(a)**, white blood cell count (WBC) **(b)**, differential WBC in relative percentages **(c) **and absolute numbers of leukocytes **(d)**, delayed type hypersensitivity (DTH) **(e)**, and time course of serum anti-Mb IgG **(f) **after treatment as in Figure 1; *n *= 8 for all groups. + *P *≤ 0.05, ++ *P *≤ 0.01 *vs*. AA/PBS; # *P *≤ 0.05, ## *P *≤ 0.01 *vs*. AA/PBS-liposomes; (*) *P *≤ 0.085 *vs*. AA/DxM-P; all Mann Whitney U-test.

Only liposomal DxM-P treatment (3 × 1 mg/kg) significantly reduced the ESR (60% and 65% reduction, respectively, *vs*. AA/PBS or AA/PBS-liposomes; Figure 5a).

#### Total and differential WBC

AA rats treated with free DxM-P showed only a partially decreased (20 to 30%) leukocytosis compared to AA/PBS (Figure 5b). In contrast, liposomal DxM-P completely reversed the leukocytosis observed in untreated AA, showing a WBC count similar to that of healthy controls (*P *≤ 0.01; significantly stronger inhibition than that achieved by PBS-liposomes, *P *≤ 0.05).

Untreated AA was characterized by a significant, relative neutrophilia (increase of polymorphonuclear neutrophilic leukocytes (PMN) from 19 to 48%) and a relative lymphopenia (decrease of lymphocytes from 77 to 48%; Figure 5c). PBS-liposomes had no effect on PMN or lymphocyte count. The absolute number of PMN was significantly decreased by liposomal DxM-P (*P *≤ 0.05 vs. AA/PBS; Figure 5d), but not by free DxM-P. Despite their clear therapeutic efficacy, however, both free DxM-P and liposomal DxM-P led to a further, significant decrease of absolute and relative lymphocyte counts (decrease to 33% and 29%, respectively; Figure 5c, d) and to an increase of the percentages of PMN (increase to 57% and 64%, respectively).

The relative reduction of lymphocytes and the relative increase of PMN were more prominent with liposomal DxM-P than with free DxM-P. The lymphocyte percentage was significantly lower (the PMN percentage higher) after free DxM-P or liposomal DxM-P than in PBS-treated AA rats (Figure 5c).

### Immune status

#### Delayed-type hypersensitivity

Little reaction was observed after i.d. injection of Mb solution in the ear. Only AA rats treated with liposomal DxM-P showed a significantly higher DTH than healthy or arthritic controls (*P *≤ 0.05; Figure 5e).

#### Anti-Mb IgG in serum

The levels of anti-Mb IgG were significantly reduced by liposomal DxM-P from Day 26 onwards, reaching healthy control levels on Day 36 (Figure 5f). No significant differences were observed between liposomal DxM-P and free DxM-P, although the decrease of serum anti-Mb IgG was somewhat more rapid after liposomal DxM-P therapy (Figure 5f).

#### Blood lymphocytes

**Figure 6:**
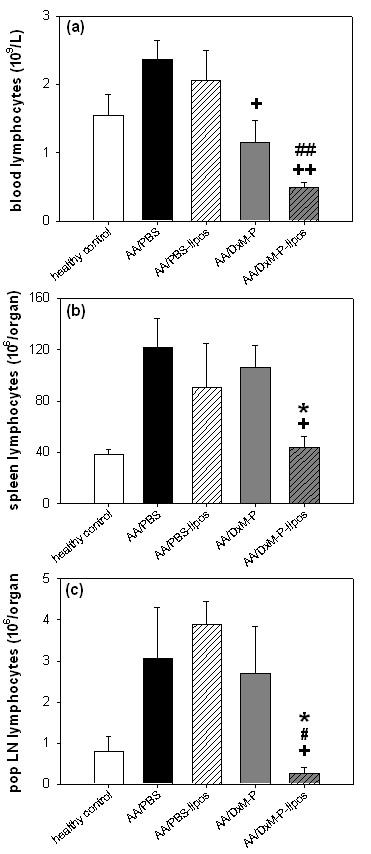
**Effects of liposomal DxM-P on lymphocytes in adjuvant arthritis (Day 21; Experiment 2/3)**. Assessment of lymphocytes in blood **(a) **(*n *= 8), spleen **(b) **(*n *= 4) and pop LN **(c) **(*n *= 4) after treatment with PBS, PBS-liposomes, free DxM-P, or liposomal DxM-P (for details see Figure 1). + *P *≤ 0.05, ++ *P *≤ 0.01 *vs*. AA/PBS; # *P *≤ 0.05, ## *P *≤ 0.01 *vs*. PBS-liposomes; * *P *≤ 0.05 *vs*. AA/DxM-P; all Mann Whitney U-test.

Free and liposomal DxM-P significantly reduced the number of circulating lymphocytes, in the case of liposomal DxM-P down to 0.49 × 10^9 ^cells/L (Figure 6a).

#### Spleen lymphocytes

In AA rats, liposomal DxM-P significantly reduced the lymphocyte numbers in the spleen to healthy control values (44 × 10^6 ^cells), whereas free DxM-P and PBS-liposomes had no significant effect on the lymphocyte infiltration (Figure 6b).

#### Pop LN lymphocytes

Only liposomal DxM-P counteracted the lymphocyte infiltration in the pop LN, leading to numbers below those of healthy controls (0.25 × 10^6 ^cells; Figure 6c). Free DxM-P had no effect.

### Cytokine production by peritoneal Mφ (PM)

**Figure 7:**
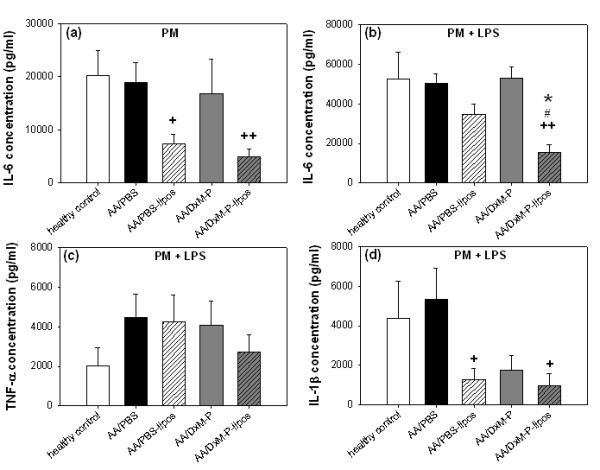
**Cytokine production by peritoneal Mφ in adjuvant arthritis (Day 21; Experiment 2/3)**. IL-6 **(a-b)**, TNF-α **(c)**, and IL-1β **(d) **production by non-stimulated and LPS-stimulated peritoneal Mφ after treatment as in Figure 1; *n *= 4 for all groups. + *P *≤ 0.05, ++ *P *≤ 0.01 *vs*. AA/PBS; # *P *≤ 0.05 *vs*. AA/PBS-liposomes; * *P *≤ 0.05 *vs*. AA/DxM-P; all Mann Whitney U-test.

In non-stimulated PM (Figure 7a) or stimulated PM (Figure 7b-d), the cytokine production did not significantly differ between healthy and PBS-treated AA rats (data only shown for IL-6).

#### IL-6

In non-stimulated PM, previous *in vivo *treatment with PBS-liposomes or liposomal DxM-P significantly reduced the IL-6 production (Figure 7a). In contrast, in LPS-stimulated PM, only liposomal DxM-P significantly reduced the IL-6 production. These effects were significantly stronger than those observed with free DxM-P (Figure 7b).

#### TNF-α

No effects of any *in vivo *therapy on TNF-α secretion were observed in non-stimulated PM (data not shown) or LPS-stimulated PM (Figure 7c).

#### IL-1β

No significant effects were observed in non-stimulated PM (data not shown). IL-1β secretion in LPS-stimulated PM was significantly reduced by *in vivo *treatment with PBS-liposomes or liposomal DxM-P (Figure 7d; free DxM-P failed to reach statistical significance).

### Pharmacokinetics/biodistribution of DxM

DxM was measured as the pharmacologically relevant species in plasma and tissues, since the pro-drug DxM-P is rapidly converted in body fluids.

#### Plasma

**Figure 8:**
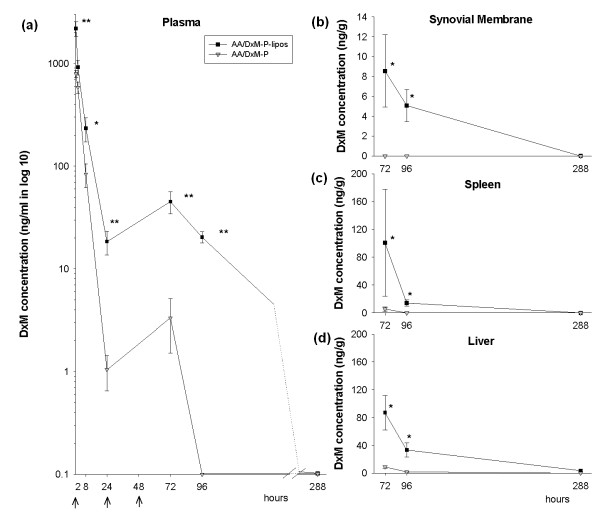
**Biodistribution of free or liposomal DxM-P in adjuvant arthritis**. Concentration of DxM in blood **(a) **(*n *= 6), synovial membrane **(b) **(*n *= 3), spleen **(c) **(*n *= 3), and liver **(d) **(*n *= 3) at 0.5, 2, 8, 24, and/or 72, 96, and 288 hours after the initial i. v. treatment with free or liposomal DxM-P during the time course of AA (for details see Figure 1). * *P *≤ 0.05, ** *P *≤ 0.01 *vs*. AA/DxM-P; all Mann Whitney U-test; administration of free or liposomal DxM-P at the time points 0 hours, 24 hours, and 48 hours.

DxM concentrations in the circulating blood rapidly decreased within the first 24 hours after injection of either liposomal or free DxM-P. However, plasma concentrations following the injection of liposomal DxM-P remained above 20 ng/mL (50 nM) throughout the 96 hours time point, significantly higher than those after injection of free DxM-P (approximately 2.8-fold at 0.5 hours; 17.7-fold at 24 hours; 14-fold at 72 hours, and ≥10-fold at 96 hours; all *P *< 0.01; Figure 8a).

#### Synovial membrane, spleen, and liver

No DxM was detectable in the synovial membrane of AA rats treated with free DxM-P at any time point investigated, while use of liposomal drug resulted in deposition of DxM at 72 hours and 96 hours (Figure 8b). Also, DxM concentrations in both spleen and liver were significantly higher 72 hours and 96 hours after treatment with liposomal DxM-P (*P *< 0.05, Figure 8c, d).

After 288 hours (nine days), the levels of DxM in almost all of the samples were below the quantification limit (2.0 ng/ml in plasma or 2.5 ng/g in tissues).

The present pharmacokinetic analysis focused on absolute concentrations of DxM as the pharmacologically relevant analyte. However, in selected spleen samples, DxM-P was also detected using a modification of the analytical procedure (LC followed by detection in the negative ionization mode). DxM-P in the spleen showed average levels of approximately 100 ng/g and 10 ng/g tissue at 72 hours and 96 hours, respectively, following injection of liposomal DxM-P, that is, similar to those of the corresponding DxM.

## Discussion

Intravenous therapy with liposomal DxM-P in established AA (analogous to the clinical situation in RA) suppressed joint swelling and inflammation in a significant, dose-dependent, and long-lasting manner. Notably, liposomal DxM-P showed superior therapeutic efficacy in both early and advanced disease compared to matched doses of free DxM-P. Liposomal encapsulation, therefore, clearly potentiates the clinical efficacy of the well-established anti-rheumatic glucocorticoid (GC) DxM-P.

This superior efficacy is already achieved upon short-term treatment for three days, similar to successful, long-term suppression of human RA [37] and rat antigen-induced arthritis (including an approximately 80% reduction of a flare-up induced seven days after the end of treatment) [19]. The prolonged efficacy may represent a substantial advantage in the treatment of RA, with the potential to obtain therapeutic effects with lower and/or less frequent doses. Of interest, the AUC for arthritis score/paw volume was more markedly reduced by liposomal than by free DxM-P, suggesting a decrease of the *cumulative *disease activity altogether.

Pharmacokinetic/biodistribution results showed a slower elimination of the liposomal drug from the circulation, confirming previous results [38], and substantially higher levels and longer persistence of the liposomal form of the drug in synovial membrane, spleen and liver until at least 48 hours after the last injection. These depot effects likely contribute to the superior and prolonged clinical efficacy of the encapsulated formulation. Enhanced therapeutic efficacy extended until Day 9 post injection of the first dose (corresponding to Day 23 of AA; Figure 1), although no DxM was detectable in blood or synovial tissue at this time point. Typical GC-receptor mediated drug actions require 1 nM concentrations or less of DxM, a level that is below the quantification limit of 5 nM (2 ng/ml) of the LC-MS method employed. Residual liposomal drugs in circulation and/or tissues may thus contribute to the persistence of clinical effects [19].

In an independent arthritis study, administration of one dose of liposomal DxM-P (1 mg/kg) and three doses of free DxM-P (3 × 1 mg/kg) showed comparable therapeutic efficacy. In addition, long-term suppression of AA by three medium doses of liposomal DxM-P (3 × 0.1 mg/kg) was basically equivalent to that of three high doses of free DxM-P (3 × 1 mg/kg). Furthermore, blood DxM concentrations 24 hours after a single dose of liposomal administration (1 mg/kg) were 17-fold and 5-fold higher, respectively, than 24 hours after a single or triple administration of free drug (one or three doses of 1 mg/kg). Thus, the present results indicate that liposomal encapsulation allows a dose reduction by a factor of at least 3, if not 10. Also, the difference between the clinical efficacy of matched doses of liposomal and free DxM-P was augmented with rising DxM-P concentrations, suggesting that potentiation by liposomal encapsulation may be enhanced by a higher dose of DxM-P.

Dose reductions via encapsulation have been recently described in AA with prednisolone-containing PEGylated-liposomes [22,23]. Avnir *et al*. [23] achieved an effective suppression of arthritis by administering two or three doses of 10 mg/kg methylpredisolone either in early AA (Days 10 and 14, or Days 10, 14 and 18) or in established AA (Days 19 and 23) using PEGylated, remote-loaded liposomes. The latter results are very similar to those of the present study, in particular when considering the differences between methylprednisolone and DxM in terms of glucocorticoid equivalent. To our knowledge, however, this study provides the first evidence of a prolonged and nearly 100% remission in severe established AA with DxM-P in non-PEGylated liposomes.

The long-term benefit of short-term therapy with liposomal DxM-P was histologically confirmed by significant reduction of inflammation, as well as bone and cartilage destruction. At this relatively early stage of disease (Day 21), the histological effects of free DxM-P were similar to those of liposomal DxM-P, and did not yet reflect the clinical rebound observed with free DxM-P after Day 19. Interestingly, only liposomal DxM-P accumulated in the inflamed synovial membrane, matching the targeting of the inflamed joint achieved by other novel liposome formulations [23]. Based on the known direct and indirect contribution of synovial Mφ to cartilage and bone destruction *via *TNF-α, IL-1β, or IL-6 [2,3,7,39], the present histological findings suggest that both Mφ activation and secretion of pro-inflammatory mediators are inhibited by effectively targeting these cells with liposomal DxM-P. Local efficacy of liposomal DxM-P extended to periarticular tissue (data not shown) and pop LN, in which liposomal (but not free) DxM-P reduced the number of lymphocytes presumably involved in regional perpetuation of inflammation [40].

The present study demonstrates for the first time that, in contrast to free DxM-P, i.v.-injected liposomal DxM-P showed systemic anti-inflammatory effects by profoundly decreasing ESR and total WBC during acute and chronic AA. Although leukocytosis in PBS-treated AA was already paralleled by an increase of the relative percentage of PMN and a reduction of the lymphocyte percentage, both free DxM-P and liposomal DxM-P caused a further significant decrease of circulating lymphocytes. Lymphocytes were reduced to levels below 50% and 75% of those in healthy controls by both free and liposomal DxM-P, respectively, suggesting that lymphopenia may be the result of direct DxM effects in addition to indirect effects via Mϕ inactivation.

Free GCs, in particular DxM-P, are known to diminish the number of circulating lymphocytes via two mechanisms [41]: 1) redistribution to spleen, lymph nodes, bone marrow, and perivascular compartments; 2) induction of cell apoptosis. In the present study, however, significantly lower numbers of lymphocytes in lymphoid organs were observed after therapy with liposomal DxM-P, suggesting that redistribution to spleen and LN either did not occur or was balanced by increased local lymphocyte apoptosis. Since liposomes are hardly taken up by lymphocytes *in vitro *([42] and own results, data not shown), direct induction of lymphocyte apoptosis by liposomal DxM-P is unlikely. Thus, the specific contribution of the prolonged accumulation of liposomal DxM in the spleen to systemic lymphocyte redistribution remains to be analyzed.

It is presently unclear whether the therapeutic efficacy of liposomal DxM-P is due to sustained immunosuppression. On one hand, the DTH to pathogenetic Mb was significantly increased on Day 21 after liposomal DxM-P treatment when compared to AA/PBS, which speaks against immunosuppression. This may indicate a shift of the Th1/Th2 balance [40,43] in favor of a protective T cell reactivity, for example by modulating the Mφ cytokine pattern [44] and/or by inducing a *regulatory *Th1 type DTH [45]. On the other hand, liposomal (but also free) DxM-P completely reverted the pathologically increased levels of specific serum anti-Mb IgG after Day 21; thus, the therapeutic effect of liposomal DxM-P may at least be partially due to, or associated with, effects on humoral immunity. However, since total IgG was not evaluated, it remains unclear whether this represents broad suppression of the humoral immune response or rather selective inhibition of disease-triggered immunity. The delay of the effects on the specific IgG levels compared to those on the cell-mediated immunity is likely due to the long half-life of the circulating IgG.

Mφ were clearly addressed by the liposomal DxM-P, as shown by a significant, persistent inhibition of the secretion of pro-inflammatory cytokines in peritoneal Mφ (until five days after the end of therapy). At this point in time, liposomal DxM-P had little effect on TNF-α secretion by peritoneal Mφ, suggesting that, if these results are transferable to the arthritic joints [40], clinical improvement may not be primarily mediated by local reduction of TNF-α, but rather by reduction of IL-1β and IL-6.

As described above, clinical efficacy of liposomal DxM-P may be related to its prolonged circulation in the blood and its enhanced accumulation in the affected joint; however, marked accumulation in other immunologically active organs (spleen and liver) is also likely to influence AA on a local and systemic level. A similar marked accumulation was observed in the spleen of AIA rats following injection of our liposomal DxM-P [19]; thus, the spleen may represent a key efficacy target of these novel liposomes, in line with the known contribution of the spleen to inflammation severity in acute AA [40]. On the other hand, liver acumulation may reduce the number of activated macrophages in the liver and thereby suppress the subsequent local production of pro-inflammatory cytokines such as IL-1β [46]. However, in addition to local and systemic depot effects of liposomal DxM, also non-genomic effects of high DxM peak levels may contribute to its therapeutic efficacy [38].

The administration of three doses of 1 mg/kg liposomal DxM-P transiently reduced the body weight. This is a known, rat-specific side-effect of glucocorticoids (observed at doses above 0.05 mg/kg DxM), mediated by decreased ribosomal amino acid incorporation into muscle cells [47], and possibly related to low plasma levels of released DxM-P [20]. Thus, lymphopenia (see above) and body weight reduction appeared to be the only evident side-effects of treatment. Of note, higher drug adsorption of liposomal DXM-P in tissues and organs is not associated with stronger GC side effects. Instead, triggering of the HPA axis, gluconeogenesis or lymphocyte alterations are markedly reduced in comparison with the effects of free DxM-P [38].

Drug-free liposomes showed a modest pro-inflammatory effect in acute and chronic AA (especially after Day 26). PEGylated liposome formulations are known to induce allergic reactions [26,48], possibly as a result of complement activation [49]. However, liposome size (especially above 90 nm) can also play a key role in complement activation [50], potentially explaining the slightly increased lymphocyte number in pop LN observed with the non-PEGylated PBS-liposomes in our study (mean size 300 nm). Pro-inflammatory effects in the arthritic joint, in turn, may be due to increased liposome uptake by activated Mφ, possibly triggered by lysosomal degradation of DPPC to lyso-phosphatidylcholine, which significantly increases Mφ ingestion [51]. Recent improvements such as the increase of the drug-to-lipid ratio may allow diminishing the lipid load [23].

## Conclusions

The present investigation on this Mφ-targeting formulation is a good example of translational research and shows that encapsulation of potent anti-inflammatory steroids has the potential to enhance/prolong therapeutic efficacy and limit side-effects. This preparation may therefore be suitable to obtain long-lasting effects with lower or less frequent dosages of GC in non-immunogenic drug carriers for the treatment of human diseases such as RA. Depot and/or recirculation effects in plasma, inflamed joint, liver, and spleen may contribute to the enhanced efficacy of liposomally encapsulated DxM-P.

## Abbreviations

AA: adjuvant arthritis; AcCN: acetonitrile; Ab: antibody; AUC: area under the curve; DMARD: disease modifying anti-rheumatic drug; DPPC: 1,2-dipalmitoyl-sn-glycero-3-phosphocholine; DPPG: 1,2-dipalmitoyl-sn-glycero-3-(phosphor-rac-(1-glycerol))(sodium salt); DTH: delayed type hypersensitivity; DxM-P: dexamethasone phosphate; ESI: electrospray ionization; ESR: erythrocyte sedimentation rate; FA: formic acid; GC: glucocorticoids; HE: haematoxilin & eosin; HPLC: high performance liquid chromatography; i.d.: intradermal; IL: interleukin; Ig: immunoglobulins; IS: internal standard; i.v.: intravenous; LC: liquid chromatography; LPS: lipopolysaccharide; LN: lymph node; Mb: Mycobacterium butyricum; Mφ: macrophages; MS: mass spectrometry; PBS: phosphate-buffered saline; PEG: polyethyleneglykol; PM: peritoneal macrophages; PMN: polymorphonuclear neutrophilic leukocytes; RA: rheumatoid arthritis; TNF: tumor necrosis factor; WBC: white blood cell count.

## Competing interests

Steffen Panzner is founder and shareholder of Novosom AG. Novosom AG holds patent WO/2004/047792: Liposomal glucocorticoids, which is granted or pending in several jurisdictions. RB and RWK have each received a one-time compensation of <10,000 Euros as inventors.

## Authors' contributions

RA carried out the study, performed the statistical analysis and drafted the manuscript. AF, MC and FP participated in the design, coordination and execution of the *in vivo *studies. DP carried out the dose-response analysis and MG evaluated the histological slides. APS, TK, and CT designed, executed and described the pharmacokinetics/biodistribution experiments. RB coordinated dose response analysis, histological studies, and immunoglobulin ELISAs. SP and UR manufactured the liposomes and participated in the drafting of the manuscript. RWK initiated the study, participated in its design and coordination, and was the main contributor to the manuscript. All authors read and approved the final manuscript.

## Authors' information

This study represents the first step of investigations carried out on this novel glucocorticoid formulation, in which its clinical efficacy has been proven. Molecular effects and mechanism of actions on Mφ have also thoroughly been investigated in a separate series of *in vitro *experiments and are the subjects of a separate manuscript.
